# Functional Analysis of IRF1 Reveals its Role in the Activation of the Type I IFN Pathway in Golden Pompano, *Trachinotus ovatus* (Linnaeus 1758)

**DOI:** 10.3390/ijms21072652

**Published:** 2020-04-10

**Authors:** Ke-Cheng Zhu, Nan Zhang, Bao-Suo Liu, Liang Guo, Hua-Yang Guo, Shi-Gui Jiang, Dian-Chang Zhang

**Affiliations:** 1 Key Laboratory of South China Sea Fishery Resources Exploitation and Utilization, Ministry of Agriculture and Rural Affairs, South China Sea Fisheries Research Institute, Chinese Academy of Fishery Sciences, Guangzhou 510300, China; zhukecheng@scsfri.ac.cn (K.-C.Z.); 398730316@163.com (N.Z.); liubaosuo343@163.com (B.-S.L.); zsdxgl@163.com (L.G.); guohuayang198768@163.com (H.-Y.G.); jiangsg@21cn.com (S.-G.J.); 2 Guangdong Provincial Engineer Technology Research Center of Marine Biological Seed Industry, Guangzhou 510300, China; 3 Tropical Aquaculture Research and Development Center, South China Sea Fisheries Research Institute, Chinese Academy of Fishery Sciences, Sanya 572018, China; 4 Sanya Tropical Fisheries Research Institute, Sanya 510300, China

**Keywords:** *Trachinotus ovatus*, IRF1, type I IFN, mutation analyses, EMSA

## Abstract

Interferon (IFN) regulatory factor 1 (IRF1), a transcription factor with a novel helix–turn–helix DNA-binding domain, plays a crucial role in innate immunity by regulating the type I IFN signaling pathway. However, the regulatory mechanism through which IRF1 regulates type I IFN in fish is not yet elucidated. In the present study, IRF1 was characterized from golden pompano, *Trachinotus ovatus* (designated ToIRF1), and its immune function was identified to elucidate the transcriptional regulatory mechanism of *ToIFNa3*. The full-length complementary DNA (cDNA) of *IRF1* is 1763 bp, including a 900-bp open reading frame (ORF) encoding a 299-amino-acid polypeptide. The putative protein sequence has 42.7–71.7% identity to fish IRF1 and possesses a representative conserved domain (a DNA-binding domain (DBD) at the N-terminus). The genomic DNA sequence of *ToIRF1* consists of eight exons and seven introns. Moreover, *ToIRF1* is constitutively expressed in all examined tissues, with higher levels being observed in immune-relevant tissues (whole blood, gill, and skin). Additionally, *Cryptocaryon irritans* challenge in vivo increases ToIRF1 expression in the skin as determined by Western blotting (WB); however, protein levels of ToIRF1 in the gill did not change significantly. The subcellular localization indicates that *ToIRF1* is localized in the nucleus and cytoplasm with or without polyinosinic/polycytidylic acid (poly (I:C)) induction. Furthermore, overexpression of ToIRF1 or ToIFNa3 shows that *ToIRF1* can notably activate *ToIFNa3* and interferon signaling molecule expression. Promoter sequence analysis finds that several interferon stimulating response element (ISRE) binding sites are present in the promoter of *ToIFNa3*. Additionally, truncation, point mutation, and electrophoretic mobile shift (EMSA) assays confirmed that *ToIRF1* M5 ISRE binding sites are functionally important for *ToIFNa3* transcription. These results may help to illuminate the roles of teleost *IRF1* in the transcriptional mechanisms of type I IFN in the immune process.

## 1. Introduction

Interferon (IFN) regulatory factors (IRFs) are important transcriptional modulators of bacteria-, parasite-, virus-, and IFN-induced signaling pathways in the process of response to virus infection, immune response, cell growth, and apoptosis [1,2,3,4,5]. To date, 10 members of the IRF family were described in higher vertebrates [6,7,8]: nine IRFs (IRF1/2/3, IRF4/ICSAT/Pip, IRF5/6/7, IRF8/ICSBP, and IRF9/ISGF3g/p48) were identified in *Mus musculus* and *Homo sapiens*, while IRF10 is observed only in avian and fish species. Moreover, IRF11 can be found in lower vertebrates, such as *Danio rerio* [9], *Miichthys miiuy* [10], and *Epinephelus coioides* [11]. In the N-terminal region of IRFs, six tryptophan repeats, which are beneficial for binding to interferon stimulating response element motifs (ISREs, GAAANNGAAA) in the promoter of target genes, are characterized in the conserved DNA-binding domain (DBD) [2,12]. Furthermore, it was reported that the IRS consensus sequence (AANNGAAA), which exists in the promoter region of IFN-β, could be bound by IRF family members [13]. In the C-terminal region of IRFs, most IRFs share an IRF3 superfamily domain, which was also named IRF-associated domain 1 (IAD1); however, IRF1 and IRF2 do not possess conserved IAD1. IAD2 could mediate protein–protein interactions instead of IAD1 [14,15]. Depending on the multifarious function, IRFs are divided into three groups: activators (IRF1/3/9), repressors (IRF8), and bifunctional ones that both activate and inhibit gene transcription relying on target genes (IRF2/4/5/7); they are classified by a regulatory region, the less-conserved C-terminal domain [16,17,18,19]. The properties of IRFs to perform diverse functions in IRF-dependent gene regulation are determined by the structural features of DBD and IAD.

According to the number of cysteine residues, IFNs are divided into two major categories. Type I IFNs contain two cysteine residues in all teleost fish lineages; nevertheless, type II IFNs include four cysteine residues in only a few species [19,20,21,22]. The transcriptional activation of type I and II IFNs is regulated by IRFs in both vertebrates and invertebrates [23,24,25]. In mammals, IRF1 was first discovered to bind and activate the IFNβ promoter [26]. Additionally, *IFNɑ* expression is mediated by IRF1 and IRF2 in leukemia patients [27]. In lower vertebrates, type I IFNs are activated by IRF2 and IRF5 in *Trachinotus ovatus* [28,29]. In invertebrates, amphioxus *Branchiostoma belcheri tsingtauense* IRF1 was found to bind ISRE and recognize the promoter of human IFNα1, IFNα2, IFNα6, and IFNβ [30]. Pacific oyster *Crassostrea gigas* IRF1 could bind ISRE, regulate the expression of IFNLP as a transcriptional regulatory factor, and participate in the antiviral immune response of oysters [23]. Consequently, IRF1 is a vital transcription factor that regulates the expression of type I and II IFN. However, the signaling pathways and biological function of *IRF1* are not yet characterized in detail in teleosts.

*T. ovatus* (Carangidae, Perciformes), which is distributed in the Asia Pacific region, is considered an important aquaculture fish in South China [31,32]. Marine white spot disease, caused by *Cryptocaryon irritans* [33], is a dominant threat to *T. ovatus* [34]. The lifecycle of *C. irritans* spans four important periods, i.e., trophont, protomont, tomont, and theront [33]. The infection seriously damages the physiological functions of the gills and skin, which are the infection site of the host [35]. To date, the disease caused considerable economic losses, estimated to be several hundred million dollars [36]. Moreover, some research showed that IRF1 can be upregulated after challenge with different types of bacteria, viruses, or polyinosinic/polycytidylic acid (poly (I: C)) in fish [37,38,39,40,41], suggesting that IRF1 plays a role in host antiviral and antibacterial responses. These findings raise the question of whether IRF1 plays a similar role in antiparasitic diseases in *T. ovatus*. Bioinformatic analysis revealed several ISRE sites in the promoter of IFNα3 in *T. ovatus*, but it is unclear whether *T. ovatus IFNα3* (*ToIFNα3*) is involved in antiparasitic diseases and how IRF1 plays its regulatory role. Therefore, to investigate the potential function of *ToIFNα3* and transcriptional regulation of ToIRF1, the present study focused on describing the importance of ToIRF1 in the regulation of *ToIFNα3* expression. Firstly, overexpressed ToIRF1 was used to detect the transcription of type I IFN signaling molecules. Secondly, to demonstrate whether IRF1 was the key element in the *ToIFNα3* promoter, promoter activity assays employing mutations to potential IRF1 binding sites were implemented. Finally, the role of the IRF1 M5 binding site in the *ToIFNα3* promoter was explored using an electrophoretic mobility shift assay (EMSA). These results may help to characterize the regulation associated with type I IFN-associated signaling in marine fish.

## 2. Results

### 2.1. Sequence Characterization of ToIRF1

**Figure 1:**
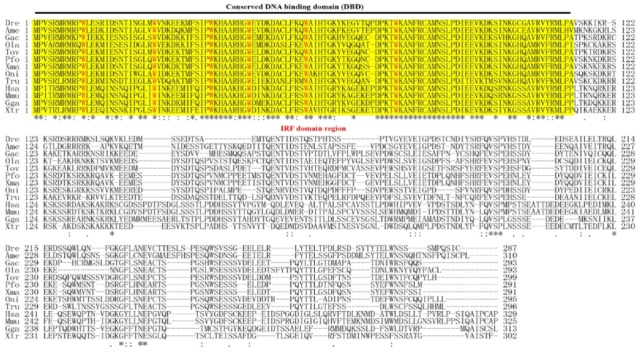
Amino-acid sequences of interferon (IFN) regulatory factor 1 (IRF1) homologs in vertebrates. The overline indicates the conserved DNA-binding domain (DBD) signature (amino acids (aa) 1–113), which contains six conserved tryptophan residues. It is also called the IRF domain (yellow underlay). Identical (asterisks) and similar (: or ∙) residues identified by the CLUSTAL W program are indicated. The Latin abbreviation and accession numbers are listed in Table S1 (Supplementary Materials).

The full-length complementary DNA (cDNA) of *ToIRF1* (GenBank accession number: MN244166, Figure S1) is 1763 bp, containing a 135-bp 5′-untranslated region (5′-UTR), a 728-bp 3′-UTR, and a 900-bp (299-amino-acid (aa)) open reading frame (ORF) with an assumed molecular weight (Mw) of 34.65 kDa and theoretical isoelectric point (pI) of 4.94. An ordinary messenger RNA (mRNA) instability motif (ATTTA) and polyadenylation signals (AAUAAA) are observed 199 bp and 15 bp upstream of the poly (A) tail in the 3′-UTR, respectively. Moreover, the multiple sequence alignment shows that the predicted aa have a winged-helix conserved DNA-binding domain (DBD, Met^1^–Val^107^) that is located in the N-terminal region and includes six typical tryptophan residues (Trp^11^, Trp^26^, Trp^38^, Trp^46^, Trp^58^, and Trp^76^) (Figure 1, Figure S1). Additionally, the deduced total aa and DBD sequence of ToIRF1 shares 42.7–71.7% similarity and 69.0%–95.6% identity with that of other IRF-1 species, respective to total aa and DBD sequence, such as 71.7% homology identity with *Oreochromis niloticus* IRF1 in total aa and 95.6% homology identity with *Poecilia formosa* IRF1 in the DBD domain (Figure 1, Table S1).

### 2.2. ToIRF1 Structural and Phylogenetic Analysis

**Figure 2:**
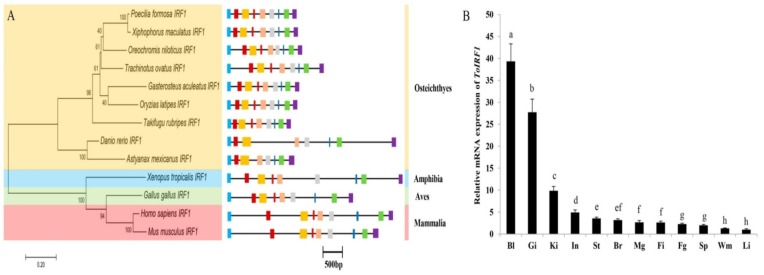
Evolutionary status, structure, and tissue expression of the *ToIRF1* gene. (**A**) Genome structure analysis of IRF1 genes according to the phylogenetic relationship. Lengths of exons and introns of each IRF1 gene are displayed proportionally. Different colored boxes and lines represent exons and introns, respectively. The identical colored boxes represent homologous sequences. (**B**) Gene transcription of ToIRF1 in various tissues. The 12 tissues are whole blood (Bl), gill (Gi), head-kidney (Ki), small intestine (In), stomach (St), brain (Br), male gonad (Mg), fin (Fi), female gonad (Fg), spleen (Sp), white muscle (Wm), and liver (Li). Different letters indicate significant differences.

The genomic sequence of *ToIRF1* is 3220 bp, including nine exons and eight introns (Figure 2A, Table S2). The typical sequence characteristics (GT/introns/AG) are also displayed in all the 5′/3′-ends of the introns [42]. The lengths and distributions of the genomic organization of metazoan *IRF1* genes are shown in Table S2. At present, *IRF1* has an analogous exon/intron structure in vertebrates from lower vertebrates (fish) to higher vertebrates (mammals) with nine exons and eight introns, except for eight exons and seven introns in both *D. rerio* and *Astyanax mexicanus*. Additionally, the phylogenetic relationship of IRF1 was determined in vertebrates (Figure 2A). The phylogenetic tree of IRF1 aa was constructed by Clustal W alignment and MEGA 6.0 software with a maximum likelihood (ML) method. In the topological structure, *ToIRF1* is grouped together with *O. niloticus* IRF1. From near to far, the homology with ToIRF1 is as follows: Osteichthyes, Amphibia, Aves, and Mammalia (Figure 2A). This result is in accordance with the traditional taxonomic relationship of the above species.

### 2.3. Tissue Expression of ToIRF1

To investigate the role of *ToIRF1* in various tissues, the constitutive expression of *ToIRF1* mRNA in the whole blood, gill, head-kidney, small intestine, stomach, brain, male gonad, fin, female gonad, spleen, white muscle, and liver was detected by qRT-PCR (Figure 2B). The highest expression of *ToIRF1* was in the whole blood, gill, and head-kidney, while the lowest mRNA levels were observed in the white muscle and liver (*p* < 0.05).

### 2.4. Protein Expression Pattern after C. irritans Infection

**Figure 3:**
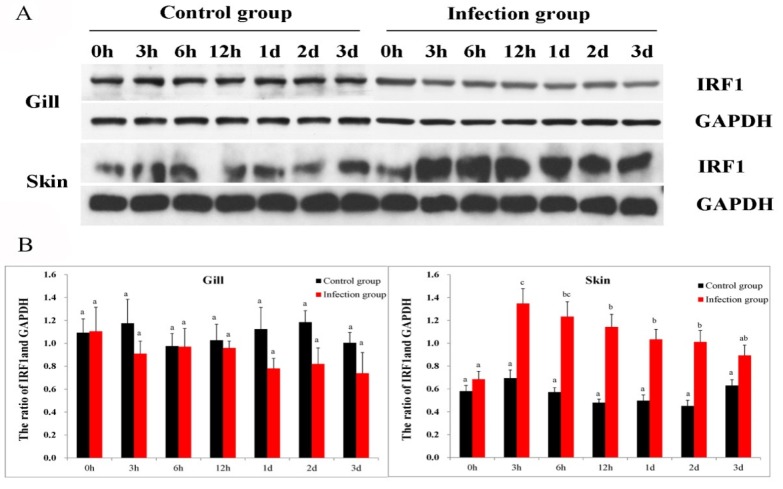
Western blot analysis of ToIRF1 proteins in gill and skin after infection with *Cryptocaryon irritans* (0, 3, 6, 12 hpi, 1 d, 2 d, and 3 d) in *Trachinotus ovatus*. (**A**) Western blot analysis was used to detect ToIRF1 expression. The experiment was divided into two groups, the control and infection groups. Lines 1 and 3 indicate the protein levels of ToIRF1 in gill and skin, respectively. Lines 2 and 4 indicate the levels of reference protein. (**B**) The corresponding ratio of gray values of ToIRF1 and GAPDH proteins; bars on the same group with different letters are significantly different from one another (*p* < 0.05).

To analyze the possible role of ToIRF1 in the defense against parasite infection, the protein levels of ToIRF1 were determined in local infection sites (skin and gills) after *C. irritans* challenge by Western blot analysis. Glyceraldehyde-3-phosphate dehydrogenase (GAPDH) was used as an internal control for normalization. The expression pattern of ToIRF1 protein was stable throughout the infection (Figure 3). Moreover, in the skin, ToIRF1 also positively responded after *C. irritans* abduction. ToIRF1 expression was significantly increased during the period of 3 h to 2 d after infection, and the peak ToIRF1 protein level was at 12 h (Figure 3). Overall, *C. irritans* stimulation in vivo can only upregulate ToIRF1 expression in skin.

### 2.5. Cytosol and Nucleus Distribution of ToIRF1

**Figure 4:**
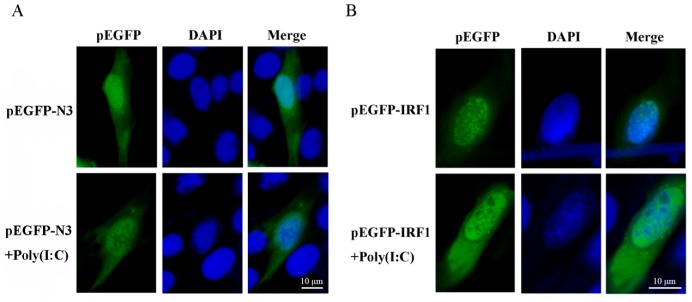
Subcellular localization of ToIRF1 in pompano cells. golden pompano snout tissue (GPS) cells seeded onto microscopy cover glass in six-well plates were transfected with 2 μg of pEGFP-N3 or pEGFP-ToIRF1 plasmid, which were considered as the control (**A**) and experimental (**B**) group, respectively. After 24 h, the cells were stimulated with polyinosinic/polycytidylic acid (poly (I:C)) (5 µg/mL) for 12 h, and then the cells were fixed and subjected to confocal microscopy analysis. Green staining represents the ToIRF1 protein signal (**B**), and blue staining indicates the nucleus region. All experiments were repeated at least three times, with similar results.

To observe the subcellular localization of *ToIRF1*, plasmids expressing pEGFP-ToIRF1 and pEGFP-N3 (control group) were transfected into GPS cells. Subsequently, those cells were infected with poly (I:C) before use for the immunofluorescence staining assay. The fluorescent signals of pEGFP-N3 were distributed throughout the cytosol and nucleus with or without poly (I:C) stimulation (Figure 4A). Furthermore, without poly (I:C) stimulation, green signals of *ToIRF1* were also observed in the nucleus and cytoplasm, which is consistent with the results after poly(I:C) challenge (Figure 4B). On the whole, these findings clearly suggest that ToIRF1-expressing regions (green) are in the whole cell, and the distributions of *ToIRF1* are not influenced by poly(I:C) infection, suggesting that *ToIRF1* is a broadly expressed protein.

### 2.6. Ectopic Expression of ToIRF1 Positively Promotes ToIFNa3 Expression and Interferon Immune Response

**Figure 5:**
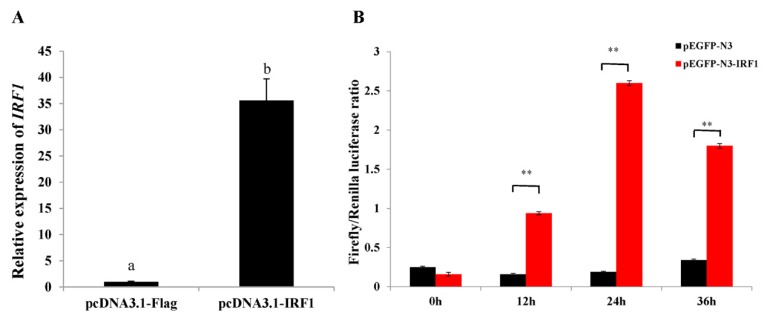
Ectopic expression of ToIRF1 increased the expression of the *ToIFNɑ3* gene. (**A**) GPS cells were transfected with ToIRF1 and empty vector, and then cells were collected for RNA extraction and qRT-PCR. (**B**) Dual-luciferase activity was driven by the ToIFNa3-p1 sequence upon the transfection of pEGFP-ToIRF1 and pEGFP-N3 into GPS cells. All values are presented as the means ± SD (*n* = 3). Asterisks indicate that the values are significantly different from the individual controls (* *p* < 0.05, and ** *p* < 0.01).

To elucidate the potential effects of *ToIRF1* overexpression on ToIFNa3 and the interferon signaling pathway, GPS cells were transfected with pcDNA3.1-IRF1 or pcDNA3.1. Using qRT-PCR, *ToIRF1* dramatically activated *ToIFNa3* expression (Figure 5A). Moreover, using luciferase reporter assays, we demonstrated that *ToIRF1* overexpression increased the promoter activity of ToIFNa3-p1 at all tested time points in GPS cells, and the maximum difference occurred at 24 h post-transfection, which was 13.7-fold higher in ToIRF1-overexpressing cells than in controls (Figure 5B).

**Figure 6:**
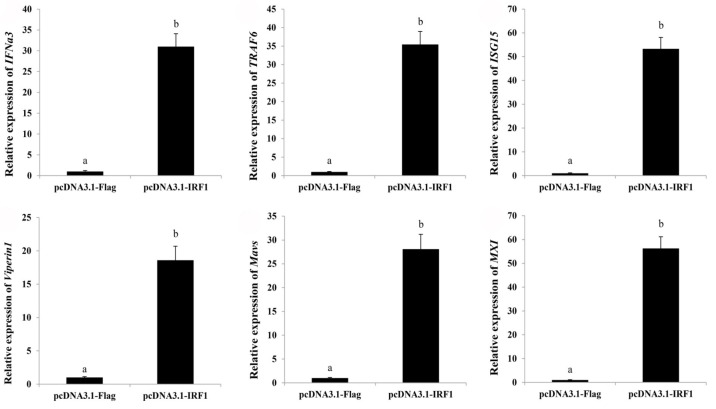
Overexpression of ToIRF1 altered the expression levels of interferon signaling molecules in GPS cells for 36 h. The expression levels of interferon signaling molecules, including *IFNa3*, *TRAF6*, *ISG15*, *Viperin1*, *Viperin2*, *Mavs*, and *MXI*, were examined using qRT-PCR analysis. The *EF-1α* gene was employed as an internal control. The messenger RNA (mRNA) expression level in GPS cells transfected with an empty vector was set as one-fold. Different letters indicate significant differences (*p* < 0.05).

To further dissect the potential mechanism underlying the antiviral action of *ToIRF1*, we evaluated the role of *ToIRF1* overexpression in the host interferon immune response. The expression levels of several interferon-related effectors or cytokines, including *TRAF6*, *ISG15*, *Viperin1*, *Viperin2, Mavs*, and *MXI*, were all observably increased in *ToIRF1*-overexpressing cells compared to the control vector-transfected cells (Figure 6). Taken together, these results indicate that *ToIRF1* positively regulates the interferon immune response in vitro.

### 2.7. Activation of the Type I IFN Response by T. ovatus rIFN

**Figure 7:**
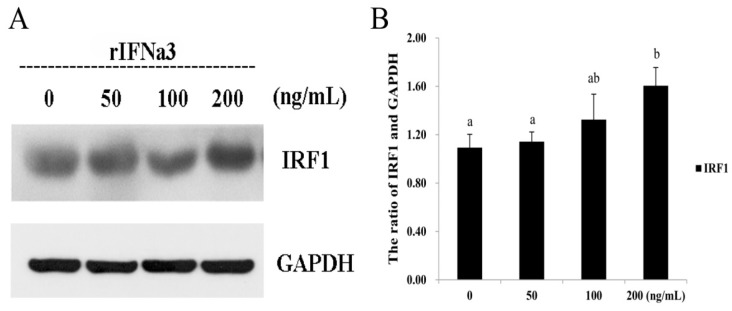
Activation of ToIRF1 in response to rIFNa3. (**A**) GPS cells were cultured in 6-cm culture dishes (2.5 × 10^6^ cells/dish) overnight and then treated with rIFNa3 in a range of doses as indicated for 24 h. GPS cell extracts were used to detect IRF1 proteins by Western blot analysis. (**B**) The corresponding ratio of gray values of ToIRF1 and GAPDH proteins. All values are presented as the means ± SD (*n* = 3). Bars on the same group with different letters are significantly different from one another (*p* < 0.05).

To investigate whether ToIFNa3 was able to activate IFN responses, GPS cells were stimulated with recombinant *T. ovatus* rIFNa3, and the protein levels of ToIRF1 were analyzed at 24 h post-induction. Western blotting showed that the protein expression of ToIRF1 was significantly increased by rIFNa3 treatment in a concentration-dependent manner (Figure 7).

### 2.8. Binding of ToIRF1 to the ToIFNa3 Sequence

**Figure 8:**
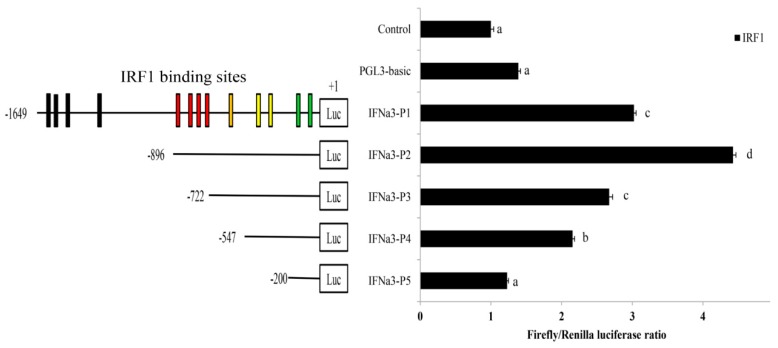
Promoter activity analysis of the *ToIFNa3* gene. The structure and transcriptional activity of *ToIFNa3* promoters. Five recombinant plasmids were constructed [28,29] and transfected with the transcription factor ToIRF1 into HEK 293T cells. Different colored boxes indicate ToIRF1 binding sites located in different truncation regions. All values are presented as the means ± SD (*n* = 3). Bars on the same group with different letters are significantly different from one another (*p* < 0.05).

To investigate the promoter activity of *ToIFNa3* in response to ToIRF1 in GPS cells, consecutive truncated mutants were constructed according to predicted IRF1-binding sites (Figure 7) [28,29]. The activity of *IFNa3-P2* is higher than the activity of other mutants with the ToIRF1 response (Figure 8), suggesting that the center region is present between −896 bp and +1 bp, which contains the IRF1 binding sites.

**Figure 9:**
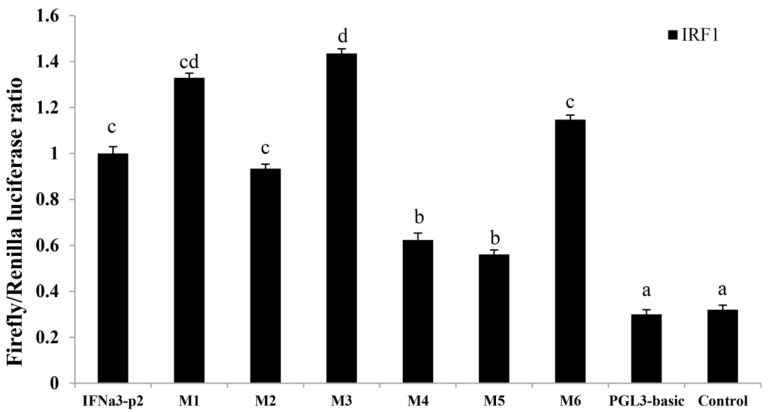
Effects of six mutants on *ToIFNa3-p2* promoter activity. Mutations of promoter sequences are according to Reference [28]. Data are presented as the means ± SD (*n* = 3). Different letters indicate significant differences (*p* < 0.05).

To further identify the ToIRF1 binding sites in the *ToIFNa3* promoter, the binding sites were deduced and mutated, as described in a previous study [28]. Endogenous cells were co-transfected with ToIRF1 and mutant vectors (M1, M2, M3, M4, M5, or M6) or the empty vector (pGL3-basic). Notably, the results show that mutations of the M4 (−496 bp to −470 bp) and M5 (−466 bp to −437 bp) binding sites caused an outstanding reduction in promoter activity (Figure 9), and no significant difference was found between the wild type (IFNa3-p2) and M1, M2, or M6. It is implied that M4 and M5 mutations are ToIRF1 binding sites in the *ToIFNa3-p2* promoter that are indispensable for triggering *ToIFNa3* expression by ToIRF1.

**Figure 10:**
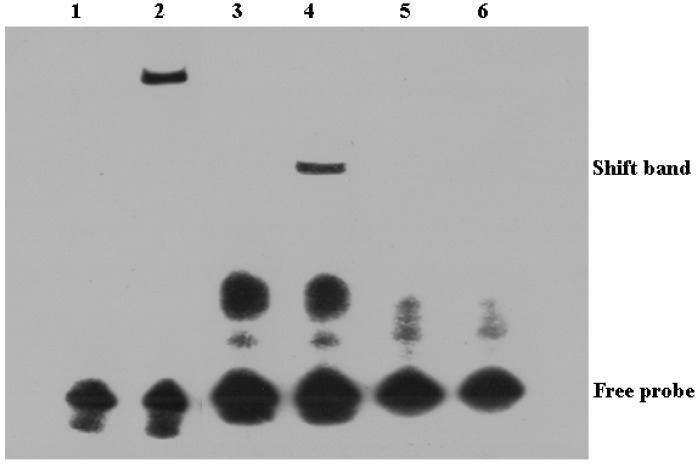
Binding reactions of IRF1 and ToIFNa3 promoter. Biotin-labeled EMSA probes were incubated with lysates of HEK293T cells containing ToIRF1 protein. WT, wild-type probe; MT: mutated probe. 1, negative control; 2, positive control; 3, plus ToIFNa3-P2-WT5; 4, ToIFNa3-P2-WT5 plus ToIRF1-Flag; 5, plus ToIFNa3-P2-MT5; 6, ToIFNa3-P2-MT5 plus ToIRF1-Flag.

To further confirm the ToIRF1 binding motif in the *ToIFNa3* promoter, an EMSA assay was implemented. Two oligonucleotide probes (IFNa3-p2-WT5 and IFNa3-p2-MUT5) were compounded and incubated with HEK293T cell lysates containing recombinant ToIRF1 in vitro according to the predicted ToIRF1 binding sites (Table 1). Recombinant IRF1 was bound to the oligonucleotide probes IFNa3-p2-WT5 and IFNa3-p2-MUT5. Mutations in nucleotides in the IRF1 binding sites resulted in the separation of the DNA-rIRF1 compound (Figure 10), suggesting that IRF1 specifically interacts with the M5 sites in the *ToIFNa3* promoter. The construction of DNA-rIRF1 compounds is specific, since it can only be blocked by excessive amounts of unlabeled control probes (100×).

## 3. Discussion

IRF1 was initially identified as a regulator that positively regulates type I IFN production and signaling and plays a pivotal role in the cellular antiviral response [43]. Subsequently, IRF1 was reported to be involved in the inhibition of cell growth and regulation of the development of NK and T cells in mammals [44]. In fish, IRF-1 also plays a key role in initiating the induction of clearance-related genes in the apoptotic cells during viral infection [45]. It is unclear whether IRF1 is involved in antiparasitic disease in fish. Moreover, the mechanism through which IRF1 regulates type I IFN in fish remains to be fully elucidated. Consequently, the present study provided insight into the mechanisms underlying the transcriptional regulation of *IFNa3* by IRF1 in *T. ovatus*. To this end, the functional characterization, tissue expression patterns, and regulatory relationship between ToIRF1 and ToIFNa3 were determined. The ToIRF1 ORFs encoded a protein that was 42.7%–71.7% identical to IRF1 proteins from other teleosts. The highly conserved DBD was in the N-terminal region, which was structurally analogous to that of Pacific oyster, *Crassostrea gigas* [23], Atlantic salmon, *Salmo salar* [38], large yellow croaker, *Pseudosciaena crocea* [39], paddlefish *Polyodon spathula* [40], half-smooth tongue sole, *Cynoglossus semilaevis* [41], orange-spotted grouper, *Epinephelus coioides* [46], and mandarin fish, *Siniperca chuatsi* [47]. Similar to other IRF1 residues in vertebrates, six tryptophan residues (Trp^11^, Trp^26^, Trp^38^, Trp^46^, Trp^58^, and Trp^76^) were located in the DBD, which was reported to polymerize a helix–turn–helix structure to bind DNA sequences containing 5′–GAAA–3′ tetranucleotides in specific promoters [1,48]. Similar to *IRF1* genes in the majority of vertebrates, the genomic sequence of *ToIRF1* is also composed of nine exons and eight introns (Figure 2A, Table S2). Nevertheless, the *IRF1* genes in *A. mexicanus* and *D. rerio* include eight exons and seven introns. Interestingly, the sizes of the third exon in *A. mexicanus* and *D. rerio* were similar to the sizes of the third and fourth exons in other vertebrates, while the sizes of other exons were highly homologous to those of exons in vertebrates. These results indicated that the genomic structure of vertebrate IRF1 was evolutionarily conserved. Moreover, phylogenetic analysis showed a typical phylogeny, revealing that the amino-acid sequences of IRF1 were closely matched to those of *O. niloticus* but then appeared to be separate from those of other fish, amphibian, avian, and mammalian species.

Previous studies demonstrated that IRF1 plays important roles in the innate immune response of vertebrates. In the present study, *ToIRF1* transcripts were broadly expressed in all tested tissues, which were consistent with the expressions of *IRF1* in the other species [23,38,39,40,41,46,47]. *ToIRF1* expression was rich in known immune-associated tissues, including whole blood, gill, and head-kidney, and poor in white muscle and liver, suggesting that ToIRF1 plays a role in the immune response. Moreover, this expression pattern of *ToIRF1* mRNA was similar to that of IRF1 in other teleosts [38,40,41,46,47]. The high expression level of *ToIRF1* was also determined in the head-kidney, which is a hematopoietic organ in teleosts. Moreover, IRF1 is also known to be a critical hematopoietic transcription factor in mammals [49]. Therefore, IRF1 may play an important role in hematopoiesis. Furthermore, the expression patterns of IRF1 in *S. salar*, *E. coioides*, *S. chuatsi*, and *P. spathula* were similar to that of IRF1 in *T. ovatus* [38,40,46,47]. This showed that IRF1 played an important role in gill tissue, a mucosa-associated lymphoid tissue in other species, but not in the *T. ovatus* [50].

*C. irritans* is considered one of the major threats limiting the development of the aquaculture industry for *T. ovatus* [34,51]. In *T. ovatus*, the parasitic sites of *C. irritans* were mainly in gill and skin [34], while *ToIRF1* transcripts were highly expressed in gill and skin. Moreover, *ToIRF2* was aggrandized in both local infection sites (gill and skin) after challenge with *C. irritans* [5]. Consequently, to explore whether IRF1 plays a key role in the resistance to *C. irritans* infection, the role of *ToIRF1* after stimulation with *C. irritans* was determined. WB results showed that similar expression levels between ToIRF1 and ToIRF2 after *C. irritans* stimulation in skin, but not in gill, implying that ToIRF1 might be involved in the host defense against *C. irritans* by mucosal immunity [5]. The peak of ToIRF1 protein level in the skin was at 3 hours post injection (hpi), and then the expressions declined. This was the difference with a previous study in Chinook salmon embryo (CHSE-214) cells [45], which showed that IRF1 was stably expressed in the infected cells from 0–48 hpi after infectious pancreatic necrosis virus (IPNV) infection. Furthermore, we did not observe significant changes in expression levels in the gill, as previously reported for other species [38,40,46,47], but this may result from the inoculation with different inducers (parasites and viruses) or different parasitic sites.

IRF1 was primarily localized to the nucleus and cytoplasm, where it retained basal transcription of a suite of antiviral genes in mammalian cells [40,52]. Consistently, *ToIRF1* was primarily situated in both the nucleus and cytoplasm with or without poly (I:C), showing that *ToIRF1* activated the downstream signaling pathway from the cytoplasm or nucleus, where *ToIRF1* was also regulated by upstream molecules. These data sustained the nuclear and cytoplasmic localization of *IRF1* as responsible for its activation and function in *T. ovatus*, revealing that the location of *IRF1* is conserved among different species [40,52].

In teleosts, according to differential structural and functional features, IFNs are divided into two subfamilies of IFNs, type I and type II IFNs [22]. The type I IFN subfamily contains a group of classic antiviral proteins, and numerous type I IFNs were authenticated [22]. As a nuclear transcription factor, an increasing number of studies indicated that IRF1 could activate IFN expression by binding the promoter sequence in mammals and fishes [37,53]. IRF1 was first characterized as a modulator of type I IFNs and IFN-inducible genes, thereby playing a vital role in innate immunity. In the present study, a positive regulatory role of ToIRF1 on *IFNa3* transcription in *T. ovatus* was proven by overexpression and promoter activity analysis. Moreover, the expression levels of several IFN/IRF-based signaling pathway genes were significantly increased in ToIRF1-overexpressing cells. These results were consistent with those in *ToIRF2-* [28], *ToIRF5-* [29], and *ToIRF8-*overexpressing [54] cells. IRF1 upregulated the expression of type I and type II IFNs, and its own expression was also regulated by both type I and type II IFNs in mammals and fish [44,45]. In the present study, ToIFNa3 treatment dramatically increased the protein levels of ToIRF1 in GPS cells. In total, we concluded that the positive modulation of the IFN/IRF-based signaling pathway by ToIRF1 might be devoted directly to its enhancing effect on immune and pro-inflammatory responses [55].

To further elucidate the binding of ToIRF1 to the *ToIFNa3* promoter sequence, the analysis of truncation mutations, point mutations, and EMSAs were implemented. The region between −896 bp and +1 bp was identified as the core regulatory region in the *ToIFNa3-p2* promoter, in which the underlying binding site of ToIRF1 was located. IRF1 bound ISRE/IRF-E motifs within IFN promoters with its DBD helix α3 to induce the transcription of IFN in *D. rerio* [56]. In *C. gigas*, IRF1 could act as a DNA-binding protein to recognize the core sequence GAAA in ISRE, rather than CAAA [23]. In the present study, deletion of the ToIRF1 M5 binding site caused prominently reduced promoter activity of *ToIFNa3* (Figure 9). The EMSA assay also showed that ToIRF1 specifically bound to the *ToIFNa3* promoter at the binding M5 site (Figure 10). ToIRF1 could bind ISRE (CAGCAGAAATCCACTGAGCGGGAAAAATAT) but not mutant ISRE (CAGCAAGAATCTGCTGAATGGGAGGAATAT) in vitro, implying that ToIRF1 could only recognize DNA sequences, including 5′–GAAA–3′ [47,48]. Briefly, ToIRF1 could control *ToIFNa3* expression by binding the M5 binding sites in fish.

In summary, the sequence, expression characteristics, and regulatory role of *ToIRF1* were described. ToIRF1 possessed representative features of the IRF family. Furthermore, the expression of *ToIRF1* was higher in immune-relevant tissues than in other tissues. The protein level of ToIRF1 was upregulated only in skin after *C. irritans* challenge in vivo, but not in the gill. Moreover, overexpression of *ToIRF1* showed that *ToIRF1* positively regulated the interferon immune response in vitro. ToIFNa3 could also positively monitor IRF1 expression in vitro. Thus, a positive feedback mechanism mediated by type I IFN-induced IRF1 activation was proposed in *T. ovatus*. Additionally, *ToIRF1* activated ToIFNa3 expression by binding with the ISRE site on its promoter. EMSA assays further verified that *ToIRF1* bound effectively to the M5 binding sites in the ToIFNa3 promoter. These findings might help to clarify the feedback regulation mechanisms of fish IRF1 and type I IFNs.

## 4. Materials and Methods

### 4.1. Ethics Statement

In the present study, all experiments were permitted by the Animal Care and Use Committee of South China Sea Fisheries Research Institute, Chinese Academy of Fishery Sciences (No. SCSFRI96-253, approval date: 23 January 2019), and the trials were implemented based on the related regulations and guidelines established by this committee.

### 4.2. C. irritans Challenge and Sampling

Healthy juvenile fish (98.0 ± 15.0 g) and senior fish (356.0 ± 25.0 g) were acquired from Linshui Marine Fish Farm in Hainan Province, China. The fish were accommodated for two weeks and fed commercial feed (Hengxin, Zhanjiang, China; crude fat >7% and crude protein >37%) before the test and were preserved in fresh seawater at 26–30 °C with 25‰ salinity. The *C. irritans* infection experiment was performed according to Zhu et al. (2020) [5]. The fish were divided into two groups: the infection group and the control group. A total of 120 fish were stimulated with *C. irritans* at a dose of 600 theronts/fish in triplicate, and 50 fish were identified as the control group. Two parasitic tissues (skin and gill) were collected after challenge for 0 h, 6 h, 12 h, 1 d, 2 d, and 3 d from six challenged fish. Moreover, the same tissues from the control group were considered to be a negative control at each time point. To investigate the tissue expression pattern of ToIRF1, adult fish tissues (*n* = 3) containing heart, eye, skin, brain, fin, spleen, small intestine, gill, white muscle, kidney, liver, stomach, and male and female gonads were sampled. Before dissection, all fish were anesthetized using MS222 (0.1 g/L; Sigma, Alcobendas, Spain). All samples were instantly frozen in liquid nitrogen and then stored at −80 °C until use.

### 4.3. RNA Isolation, cDNA Synthesis, and Protein Extraction

Total RNA was extracted from different tissues and cells with TRIzol reagent (Invitrogen, USA) following the manufacturer’s protocol. Total RNA quality and quantity were authenticated by 1% agarose gels and a NANODROP 2000 spectrophotometer (Thermo Scientific, Waltham, MA, USA), respectively. Moreover, Oligo(dT)_16_ (0.5 μg) and RNA (1 µg) were mixed and reacted for 5 min at 70 °C. Then, the mixture was refrigerated for 2 min on ice, and M-MLV (200 units), RNasin (25 units), 5× buffer, and dNTPs (0.8 mM) were added in a total volume of 25 μL and extended for 1 h at 42 °C for reverse transcription. Moreover, the total protein was extracted using the ProteoPrep® Total Extraction Sample Kit (Sigma-Aldrich).

### 4.4. Cloning of cDNA and Genomic Sequences

The *IRF1*-derived sequences were acquired from *T. ovatus* genomic data [57]. Furthermore, to clone the corresponding *ToIRF1* sequence from gill cDNA of healthy fish, gene-specific primers were designed by Primer Premier 5 (Table 1). The relevant sequences of *ToIFNa3* were referenced in our previous study [28]. A 900-bp fragment of the *IRF1* gene was obtained. This fragment was used as a core sequence to amplify the full-length cDNA of *IRF1* with the Rapid Amplification of cDNA Ends (RACE) method (Table 1). According to the manufacturer’s instructions, 5′/3′-RACE polymerase chain reaction (PCR) was implemented using the RACE cDNA Amplification Kit (TaKaRa, Japan). The PCR products of 5′/3′-RACE were cloned into the pGEM®-T Easy Vector (Promega, USA).

### 4.5. Bioinformatics

The basic local alignment search tool (BLAST) program from the national center for biotechnology information national center for biotechnology information (NCBI) database (http://blast.ncbi.nlm.nih.gov/Blast.cgi) was used to identify the amino acid (aa) sequences of *ToIRF1*. The exon and intron sequences of the *IRF1* gene were determined by Ensembl (http://asia.ensembl.org/). Different aa sequences of IRF1 were aligned by ClustalW2 (http://www.ebi.ac.uk/Tools/msa/clustalw2/). Moreover, MEGA 6.0 software was used to structure a maximum likelihood (ML) phylogenetic tree (LG +G model, bootstrap 1000) of IRF1 aa [58]. The genome features and phylogenetic tree were created with Adobe PhotoShop CS6 (Adobe, San Jose, CA) and FigTree v1.4.2 (http://tree.bio.ed.ac.uk/software/figtree/), respectively. To calculate the molecular weights (Mw) and theoretical isoelectric points (pI), Compute pI/Mw software (http://web.expasy.org/protparam/) was used.

### 4.6. Subcellular Localization

To structure the plasmid for subcellular localization, the cloning open reading frame (ORF) of *ToIRF1* and pEGFP-N3 (Clontech, USA) with the *BamH*I and *Xho*I sites (Table 1) was used to construct the recombinant expression vector pEGFP-N3-ToIRF1. To determine the subcellular distribution of *ToIRF1*, golden pompano (*T. ovatus*) snout tissue (GPS) cells were transfected with expressing plasmid *ToIRF1* (pEGFP-IRF1) or empty vector (pEGFP-N3), separately [59]. Twenty-four hours later, the cells were challenged with poly (I:C) (5 µg/mL) for 12 h. The cells were immobilized with 4% paraformaldehyde. Subsequently, 4,6-diamidino-2-phenylindole (DAPI) and fluorescence microscopy (Leica, Switzerland) were used to stain and examine the cells.

### 4.7. ToIRF1 Overexpression Promotes ToIFNa3 Expression

To construct the expression plasmid, the ORF of *ToIRF1* was inserted into the pCDNA3.1 vector (Invitrogen, USA) with the *BamH*I and *Xho*I sites (Table 1). To survey whether ToIRF1 could regulate the expression of *ToIFNa3* and interferon signaling molecules, GPS cells were transfected with pcDNA3.1-IRF1 or empty vector (pcDNA3.1), and the cells were harvested after 24 h. Total RNA was extracted as described above. Moreover, to further confirm the regulatory relationship between ToIRF1 and ToIFNa3, pGL3-basic-IFNa3-P1 was transfected into GPS cells together with pcDNA3.1-IRF1 or empty vector [28,29]. At 0 h, 12 h, 24 h, and 36 h post-transfection, the GPS cells were collected and lysed to determine luciferase activity using a microplate reader (Thermo, USA). The *Renilla* luciferase plasmid pRL-TK (Promega, USA) was defined as an internal control. 

### 4.8. Promoter Deletion Mutation and Point Mutation Analysis

To further investigate the ToIRF1 binding site in the promoter of *ToIFNa3*, the transcription factor binding site prediction (TFBS)-JASPAR database (http://jaspar.genereg.net/) and TRANSFAC^®^ and MatInspector^®^ software were used to predict the binding sites of ToIRF1 in the promoter sequences of *ToIFNa3*. According to the location of the predicted binding sites of ToIRF2, five truncated mutants from the *ToIFNa3* promoter were designed and preserved in our lab. The corresponding promoter cloning and plasmid construction were described in a previous study (Table 1) [28,29]. Five plasmids (denoted as pGL3-basic-IFNa3-p1 (−1649 to +1), pGL3-basic-IFNa3-p2 (−896 to +1), pGL3-basic-IFNa3-p3 (−722 to +1), pGL3-basic-IFNa3-p4 (−547 to +1), and pGL3-basic-IFNa3-p5 (−200 to +1)) were provided by our laboratory.

To validate the potential role of the ToIRF1 binding sites on the core *IFNa3* promoter, the predicted binding sites that include the ISREs (GAAANNGAAA) were mutated. Based on the prediction, six assumed recombinant plasmids of mutations were constructed as in previous research [28]. The pGL3-basic-IFNa3-p2 promoter was considered the wild-type promoter. The six point mutants were directly deleted to predict six binding sites: M1 (−799 bp to −772 bp), M2 (−768 bp to −749 bp), M3 (−703 bp to −682 bp), M4 (−496 bp to −470 bp), M5 (−466 bp to −437 bp), and M6 (−308 bp to −288 bp) from the wild-type promoter. The homologous TF binding site sequences were also shown in Reference [28].

### 4.9. Electrophoretic Mobility Shift Assay (EMSA)

The EMSA procedure was implemented according to a previously described procedure [28]. Briefly, for the objective of DNA/protein conjugation reactions, lysates of human embryonic kidney (HEK293T) (GeneCreate, Wuhan, China) transfected with pcDNA3.1-Flag-IRF1 were provided. According to the manufacturer′s instructions, the EMSA Probe Biotin Labeling Kit (Beyotime, Shanghai, China) was used to mark the wild-type and mutated oligonucleotides (Table 1). DNA/protein binding reactions were executed by EMSA/Gel-Shift Kit (Beyotime, China) at 25 °C. To comprehend the specificity of the DNA/protein binding reactions, competition assays were performed with 100× excessive unlabeled wild-type or mutated probes. Subsequently, the completed reactions were segregated on nondenaturing 4% PAGE gels for 20 min. A LightShift® Chemiluminescent EMSA Kit (Pierce, USA) was used to develop the proteins by the autoradiography method.

### 4.10. Quantitative Real-Time PCR and Statistical Analysis

The relative *ToIRF1* mRNA levels in the tissues and GPS cells were determined by quantitative real-time PCR (qRT-PCR). Total RNA was extracted from different tissues and cells as described above. The specific primers for the IFN signaling pathway genes *IRF1*, *IFNa3*, tumor necrosis factor (TNF) receptor associated factor 6 (*TRAF6*), interferon stimulated gene (*ISG15*), *viperin1, viperin2*, mitochondrial antiviral signaling protein (*Mavs*), MAX interactor 1 (*MXI*), and the reference gene elongation factor-1 alpha (*EF-1α*) are displayed in Table 1 [5,28,29]. The qRT-PCR procedure was implemented as previously described [60]. Relative expression was calculated by the 2^−ΔΔCT^ method [61].

### 4.11. Preparation of the IRF1 Polyclonal Antibody and Western Blotting Analysis

To manufacture the polyclonal anti-IRF1 antibody, a peculiar domain (IRF1 aa ^279–291^) of ToIRF1 was compounded from Genecreate (Wuhan, China). The PCR product was inserted into the pET-B2M vector using *Nde*I and *Xho*I restriction enzyme cutting sites. To express recombinant ToIRF1 protein (rToIRF1), *Escherichia coli* BL21 (DE3) (Novagen, Germany) was transformed with the recombinant plasmid. rToIRF1 was purified as previously described [62]. Furthermore, to produce a polyclonal antibody, white New Zealand rabbits were injected with purified rToIRF1 protein using standard methods [63]. Once generated, the polyclonal antibody was preadsorbed using *E. coli* lysate supernatants to detach inhomogeneous antibodies and was purified on a HiTrapTM Protein A HP column on an AKTAprime™ Plus system (GE Healthcare, USA).

To verify the specificity of the rabbit anti-IRF1 antibody, HEK293T cells were transfected with pcDNA3.1-IRF1 or pcDNA3.1 for 48 h. Then, the cells were harvested by centrifugation at 160× *g* for 10 min at 4 °C. Total protein was extracted as described above. Then, the total protein was electrophoresed on 12% SDS-PAGE and electrophoretically transferred to polyvinylidene fluoride (PVDF) membranes (Millipore, USA) using the PierceG2 Fast Blotter (25 V for 10 min, Pierce, Rockford, IL, USA). Western blotting (WB) analyses were carried out according to a previously described protocol [25].

The endogenous IRF1 protein expression response to *C. irritans challenge* in gill and skin was determined by WB analyses. Moreover, to detect endogenous IRF1, GPS cells were cultured in a 6-cm plate (2.5 × 10^6^) and treated with rIFNa3 at final concentrations of 50, 100, and 200 ng/mL or PBS (as a control) for 24 h. Total protein was isolated by 12% SDS-PAGE, and the membrane was transferred as described before. Primary antibodies (anti-IRF1, murine anti-Flag (Sigma-Aldrich, USA) and the loading control, the anti-glyceraldehyde 3-phosphate dehydrogenase antibody (GAPDH, Sigma-Aldrich, USA), 1:1000) were incubated with the PVDF membrane in 1% (*w*/*v*) non-fat milk in Tris-buffered saline and Tween-20 (TBST) buffer (0.1% Tween-20) for 3 h. Horseradish peroxidase (HRP)-conjugated goat anti-rabbit antibody (1:3000) was used as a secondary antibody (Sigma-Aldrich, USA) [60]. Finally, the results were detected using an electrochemiluminescence (ECL) system.

### 4.12. Statistical Analysis

All trials were performed in triplicate. SPSS 19.0 software (IBM, USA) was used to analyze the data. All values are displayed as the mean ± SD. Significant differences were calculated by one-way ANOVA tests, and *p* < 0.05 and *p* < 0.01 were considered to be significant.

## Figures and Tables

**Table 1:** Primers used for sequence cloning, deletion mutant construction, mRNA construction, and qRT-PCR [5,28,29]. RACE—Rapid Amplification of complementary DNA (cDNA) Ends; EMSA—electrophoretic mobile shift assay.

Subject and Primers	Nucleotide Sequence
**Primers for Sequence Cloning**	
IRF1-ORF-F	CGCGGATCCATGCCTGTGTCTCGGATGA
IRF1-ORF-R	CCGCTCGAGTCAGTGGAGGTATGGTTGGCA
IRF1-genome-F	GCTCTCATCGTATCGTGT
IRF1-genome-R	CCAAACTGGTACAGAGTC
IRF1-3′RACE-outer	TGATACTCAGTCTCCCTCGG
IRF1-3′RACE-inner	GTTACACAACACTGGGCT
IRF1-5′RACE-outer	GCACACAGAGCAGCGAGA
IRF1-5′RACE-inner	GAACTGTGACCCAAAGAC
**Deletion mutant construction**	
IFNa3-pF1	CGGGGTACCAAAAGACAACTGATTGTTGA
IFNa3-pF2	CGGGGTACCCTGCTACATATAAAAATGT
IFNa3-pF3	CGGGGTACCCAATGTGAAGAGGGTTCAG
IFNa3-pF4	CGGGGTACCTTTATTTTGTAAAGGTGAGTG
IFNa3-pF5	CGGGGTACCTACTGCACTGGTATCAGTACT
IFNa3-pR	CCGCTCGAGCATTGACATGATGCCTAACTCT
**Primers for qRT-PCR**	
qRT-IRF1-F	TGATACTCAGTCTCCCTCGG
qRT-IRF1-R	TCTCGCTGCTCTGTGTGC
qRT-IFNa3-F	ACACTATGGTCACTACAGCAAC
qRT-IFNa3-R	ACCTCAGTGTTTCGTATGTG
qRT-TRAF6-F	CCCTAAAGCACCCATCGC
qRT-TRAF6-R	AAGGTCACGCAGGAACTCAG
qRT-MXI-F	CATACCCTTGGGACCTGA
qRT-MXI-R	TGCTTTGGCTTTGTTGAGT
qRT-ISG15-F	TACGCTGAGTGAGACCCG
qRT-ISG15-R	GGAGGAACACCTGGATGG
qRT-Viperin1-F	GACCCGTCCAAGTCCATC
qRT-Viperin1-R	CAAAGCCACTGAAGCAAAT
qRT-Viperin2-F	CCCGAGTCCAATGAGAAGA
qRT-Viperin2-R	CGAAGCCACTAAAGCAGATG
qRT-Mavs-F	GTTTGGAGGTGCGGATGA
qRT-Mavs-R	CCTTTTCGGCTTTGCTGTA
EF1α-F	AAGCCAGGTATGGTTGTCAACTTT
EF1α-R	CGTGGTGCATCTCCACAGACT
**EMSA assays**	
IFNa3-P2-MUT5	CAGCAAGAATCTGCTGAATGGGAGGAATAT
IFNa3-P2-WT5	CAGCAGAAATCCACTGAGCGGGAAAAATAT

## Supplementary Materials

Supplementary materials can be found at https://www.mdpi.com/1422-0067/21/7/2652/s1.

## Abbreviations

IRF1	Interferon regulatory factor 1
ORF	Open reading frame
DBD	DNA-binding domain
ISREs	Interferon stimulating response elements
EMSA	Electrophoretic mobile shift assays
IFN	Interferon
IAD	IRF-associated domain
ML	Maximum likelihood
GPS	Golden pompano *T. ovatus* snout cell
TRAF6	TNF receptor-associated factor 6
ISG15	Interferon stimulated gene
Mavs	Mitochondrial antiviral signaling protein
MXI	MAX interactor 1
qRT-PCR	quantitative real-time polymerase chain reaction
TBST	Tris-buffered saline and Tween-20
