# Supplementary material for: Functional Analysis of IRF1 Reveals its Role in the Activation of the Type I IFN Pathway in Golden Pompano, Trachinotus ovatus (Linnaeus 1758)
Source: Int J Mol Sci. 2020 Apr 10;21(7):2652. doi: 10.3390/ijms21072652 (PMC7177527; doi:10.3390/ijms21072652)
Supplement: Supplementary file 1 [file ijms-21-02652-s001.pdf]

## Supplementary Figure

**Fig. S1** The full cDNA sequence and deduced amino acid sequence of *Trachinotus ovatus* IRF1. The full-length of cDNA was 1,763 bp, and 299 residues were deduced. The bold underlined letters indicated the start codon (ATG) and the stop codon (TGA). The letters in the box and italic represented the polyadenylation single sequence (AATAAA) and mRNA degradation motifs (ATTTA), respectively. The yellow indicates the DBD domain (aa 1-113), which contains six conserved tryptophan residues.

```
   1 ACTTCTGTCTCAGACTGTCTCTAGGTGGATAACACAAAGCCGAGGAGAAGTCTCTCTAGAGCAAACGAAA 70
  71 TCCAAGCGGATTATTTTTTGTGCATTTTCCTCTTAAGGATCATTGAACAAACAAAGCCCGCAAACatgcc 140
   1                                                                  M  P 2
 141 tgtgtctcggatgaggatgaggccgtggctggagaagatgatcgagtccaacaccatctcgggcctgatg 210
   3  V  S  R  M  R  M  R  P  W  L  E  K  M  I  E  S  N  T  I  S  G  L  M  25
 211 tgggtggacaaggagaagaccatgttctcaattccctggaagcatgcagctcgacacggctgggagctgg 280
  26 W  V  D  K  E  K  T  M  F  S  I  P  W  K  H  A  A  R  H  G  W  E  L  D 49
 281 acaaggatgcatgtctgttcaaacagtgggccatccacacagggaaatacgttgagggccagaactgtga 350
  50  K  D  A  C  L  F  K  Q  W  A  I  H  T  G  K  Y  V  E  G  Q  N  C  D  72
 351 cccaaagacgtggaaagccaacttccgctgtgcgatgaattcactgcctgacatagaggaggtgaaagac 420
  73  P  K  T  W  K  A  N  F  R  C  A  M  N  S  L  P  D  I  E  E  V  K  D  95
 421 aagagcatcaacaagggccaccaagccatgcgtgtgttcaggatgctgcctgccaccccaaaaactagag 490
  96 K  S  I  N  K  G  H  Q  A  M  R  V  F  R  M  L  P  A  T  P  K  T  R  D 119
 491 ataaacgaagcaaaggaaaggaagcaaagttaaggaggaaggacccaatggtaaagatggaggaagacat 560
 120  K  R  S  K  G  K  E  A  K  L  R  R  K  D  P  M  V  K  M  E  E  D  M  142
 561 ggactacagtgatactcagtctccctcggatgcatcactgccggatgagacaactcaggaaaacacagtg 630
 143  D  Y  S  D  T  Q  S  P  S  D  A  S  L  P  D  E  T  T  Q  E  N  T  V  165
 631 gacagtacagtgcacacagagcagcgagatttcatgtgcgtggcttcatctgaggttcctgagtggtctc 700
 166 D  S  T  V  H  T  E  Q  R  D  F  M  C  V  A  S  S  E  V  P  E  W  S  H 189
 701 attcagttgagattgggtctgagaccttctcaaggagcttctacgagagatttgaagtttcacctgagca 770
 190  S  V  E  I  G  S  E  T  F  S  R  S  F  Y  E  R  F  E  V  S  P  E  H  212
 771 cagcttcgacggcagctataccgacgacattgttgagatctgcgagcagctggagagagactcacaattt 840
 213  S  F  D  G  S  Y  T  D  D  I  V  E  I  C  E  Q  L  E  R  D  S  Q  F  235
 841 cagtggatgtcaagcagtgtagatggcaggggggttcctgagcaatgaaccatgcaccagtccagagagcc 910
 236 Q  W  M  S  S  S  V  D  G  R  G  F  L  S  N  E  P  C  T  S  P  E  S  R 259
 911 ggtggagtgactcctcctcagtcgacgaattagacgacatgccaagttacacaacactgggctcagactt 980
 260  W  S  D  S  S  S  V  D  E  L  D  D  M  P  S  Y  T  T  L  G  S  D  F  282
 981 tacaaattccacagacgaaatctggaacaccttttgccaaccatacctccactgaGAGCCTCAGGACGGA 1050
 283  T  N  S  T  D  E  I  W  N  T  F  C  Q  P  Y  L  H  *                 299
1051 CAGGACAGGACAATGCTTTGACATTTTGGACTAAAAAAGCTGTTTCTGCCCACCGTCTCCCCCCATCTTT 1120
1121 TCAGCTGAGTCACCCATCATCACCCCTCCAGTCTGTGACAGGCAGTGTCCACAATTTAAGCTAAAACACT 1190
1191 GCCCCTCAACTGCGTCTACTGTGCGTCTGACTCTGTACCAGTTTGGAGAGCTTTCCTCATTTCCCTCAAC 1260
1261 CTTTATGTGAAGTTATGAAGAGAAGATATGAAGTGCACTGCTGTTAAAGGACATTCTGTTCATTGTCTCT 1330
1331 GTTTTTGGCTGGTTCAAGGCATTGTGGTGCTCAGTTTTTTGTCATGTTTAGTTCTAATTGGATAGAACTG 1400
1401 TGTTTTGCTCAGGAAGGGTATTTACATGGTCATACTCAGCTATGAATACATACAAGCTAGTCAACACTTA 1470
1471 ACCATAAAGTGCAAATAGGCTCACATTCAGTGCTCTTCACATTTGATAGTAAGTCAGTAATTGTAGTGTC 1540
1541 AAATAGTTATCACATAAATCATTTATTTTTCCGTTATGTGTACCCATCAATTCTAGTTAAAGGGATTATA 1610
1611 AGACATTTTGTCAAATTATCTTCTTAACTCTCAACTTCTGGTAAAAGCGCTACTTCATTATCTGAGTTTT 1680
1681 TCTTACTTCGGGAATTGTAGAAAGTTTCTGTAAATAGTTGTGTCTGTCTGTGTGTTTTTATAAAATAAAA 1750
1751 AAAAAAAAAAAAA 1763
```

## Supplementary Table

**Table S1.** IRF1 proteins used in multiple alignment.

| Species | Abbreviation | Protein name | Total AA identity % | DBD domain identity % | No. |
|---|---|---|---|---|---|
| *Trachinotus ovatus* | Tov | IRF1 | 100 | 100 | MN244166 |
| *Gasterosteus aculeatus* | Gga | IRF1 | 65.42 | 84.07 | ENSGACT00000027655.1 |
| *Takifugu rubripes* | Tru | IRF1 | 57.75 | 81.42 | ENSTRUT00000010295.3 |
| *Oreochromis niloticus* | Oni | IRF1 | 71.72 | 92.92 | ENSONIT00000069535.1 |
| *Oryzias latipes* | Ola | IRF1 | 65.12 | 85.71 | ENSORLT00020035606.1 |
| *Poecilia formosa* | Pfo | IRF1 | 68.47 | 95.58 | ENSPFOT00000032009.1 |
| *Xiphophorus maculatus* | Xma | IRF1 | 67.80 | 92.92 | ENSXMAT00000011129.2 |
| *Danio rerio* | Dre | IRF1 | 55.74 | 85.09 | ENSDART00000158742.2 |
| *Astyanax mexicanus* | Ame | IRF1 | 55.41 | 83.04 | ENSAMXT00005051691.1 |
| *Xenopus tropicalis* | Xtr | IRF1 | 43.87 | 69.03 | ENSXETT00000030538.1 |
| *Gallus gallus* | Gga | IRF1 | 46.27 | 74.34 | ENSGALT00000002244.6 |
| *Homo sapiens* | Hsa | IRF1 | 42.72 | 70.80 | ENST00000245414.9 |
| *Mus musculus* | Msu | IRF1 | 44.88 | 70.80 | ENSMUST00000108920.8 |

**Table S2.** Lengths of exons (bp) and introns (bp) of each *IRF1* gene.

| Species | E1 | I1 | E2 | I2 | E3 | I3 | E4 | I4 | E5 | I5 | E6 | I6 | E7 | I7 | E8 |
|---|---|---|---|---|---|---|---|---|---|---|---|---|---|---|---|
| *Trachinotus ovatus* | 87 | 755 | 100 | 105 | 168 | 191 | 41 | 255 | 127 | 175 | 117 | 140 | 29 | 184 | 124 |
| *Gasterosteus aculeatus* | 87 | 376 | 100 | 84 | 168 | 103 | 41 | 147 | 127 | 101 | 117 | 127 | 29 | 121 | 118 |
| *Takifugu rubripes* | 87 | 86 | 100 | 74 | 168 | 105 | 38 | 108 | 133 | 91 | 117 | 111 | 26 | 151 | 121 |
| *Oreochromis niloticus* | 87 | 480 | 100 | 98 | 168 | 143 | 41 | 220 | 127 | 98 | 102 | 130 | 29 | 188 | 124 |
| *Oryzias latipes* | 87 | 148 | 100 | 83 | 168 | 134 | 38 | 172 | 133 | 100 | 117 | 107 | 29 | 195 | 91 |
| *Poecilia formosa* | 87 | 92 | 100 | 113 | 168 | 116 | 41 | 154 | 133 | 111 | 114 | 125 | 29 | 174 | 115 |
| *Xiphophorus maculatus* | 87 | 86 | 100 | 93 | 168 | 114 | 41 | 182 | 133 | 108 | 114 | 129 | 29 | 178 | 115 |
| *Danio rerio* | 87 | 88 | 100 | 250 | 209 | - | - | 603 | 100 | 137 | 102 | 531 | 29 | 162 | 124 |
| *Astyanax mexicanus* | 87 | 123 | 100 | 102 | 212 | - | - | 126 | 121 | 111 | 117 | 130 | 29 | 153 | 133 |
| *Xenopus tropicalis* | 87 | 599 | 100 | 379 | 177 | 206 | 47 | 110 | 115 | 510 | 120 | 1223 | 41 | 113 | 127 |
| *Gallus gallus* | 87 | 871 | 100 | 175 | 177 | 241 | 50 | 126 | 136 | 191 | 126 | 468 | 32 | 615 | 136 |
| *Homo sapiens* | 87 | 1366 | 100 | 795 | 177 | 109 | 50 | 108 | 130 | 183 | 123 | 534 | 50 | 1169 | 136 |
| *Mus musculus* | 87 | 1463 | 100 | 717 | 177 | 93 | 50 | 86 | 133 | 151 | 123 | 585 | 50 | 814 | 136 |
